# Mapping of a major QTL controlling plant height using a high-density genetic map and QTL-seq methods based on whole-genome resequencing in *Brassica napus*

**DOI:** 10.1093/g3journal/jkab118

**Published:** 2021-04-09

**Authors:** Zhixue Dong, Muhammad Khorshed Alam, Meili Xie, Li Yang, Jie Liu, M M U Helal, Junyan Huang, Xiaohui Cheng, Yueying Liu, Chaobo Tong, Chuanji Zhao, Shengyi Liu

**Affiliations:** 1 Oil Crops Research Institute of the Chinese Academy of Agricultural Sciences/The Key Laboratory of Biology and Genetic Improvement of Oil Crops, The Ministry of Agriculture and Rural Affairs, Wuhan 430062, P. R. China; 2 National Key Laboratory of Crop Genetic Improvement, National Center of Rapeseed Improvement, Huazhong Agricultural University, Wuhan 430070, P. R. China; 3 Biosystematics Group, Experimental Plant Sciences, Wageningen University and Research, Wageningen, The Netherlands

**Keywords:** *Brassica napus*, plant height, QTL-seq, high-density genetic map, QTL mapping, RNA sequencing

## Abstract

Plant height is a crucial element related to plant architecture that influences the seed yield of oilseed rape (*Brassica napus* L.). In this study, we isolated a natural *B. napus* mutant, namely a semi-dwarf mutant (*sdw-e*), which exhibits a 30% reduction in plant height compared with Zhongshuang 11-HP (ZS11-HP). Quantitative trait locus sequencing (QTL-seq) was conducted using two extreme DNA bulks in F_2_ populations in Wuchang-2017 derived from ZS11-HP × *sdw-e* to identify QTLs associated with plant height. The result suggested that two QTL intervals were located on chromosome A10. The F_2_ population consisting of 200 individuals in Yangluo-2018 derived from ZS11-HP × *sdw-e* was used to construct a high-density linkage map using whole-genome resequencing. The high-density linkage map harbored 4323 bin markers and covered a total distance of 2026.52 cM with an average marker interval of 0.47 cM. The major QTL for plant height named *qPHA10* was identified on linkage group A10 by interval mapping and composite interval mapping methods. The major QTL *qPHA10* was highly consistent with the QTL-seq results. And then, we integrated the variation sites and expression levels of genes in the major QTL interval to predict the candidate genes. Thus, the identified QTL and candidate genes could be used in marker-assisted selection for *B. napus* breeding in the future.

## Introduction

Seed yield is significantly influenced by plant architecture. One of the great successes of plant breeding occurred during the green revolution in the 1960s when dwarf loci were introduced in wheat. The introduction of dwarf loci led to the development of dwarf varieties that were resistant to lodging and had a higher yield and harvest index (Khush 2001). Rapeseed (*Brassica napus*, AACC, 2*n* = 38) is one of the most important oil crops in the world (Hu *et al.* 2017). In *B. napus*, plant height is not only directly related to plant architecture but is also an important agronomic trait related to yield, lodging, and mechanized harvesting (Bin *et al.* 2006). Previous research reported that the number of siliques after lodging was reduced by 17.5%, and the seed yield was decreased by 16.2% (Islam and Evans 1994). Through the utilization of heterosis in rapeseed, the plant height has generally increased by about 20 cm, which causes lodging risks in the late growth stage, in turn influencing the seed yield and quality traits (Foisset *et al.* 1995). An appropriate reduction of plant height in rapeseed has significantly improved mechanized breeding and lodging resistance. Therefore, excavating dwarf germplasm resources and genes is beneficial to the improvement of plant architecture.

Many agronomic traits of crops, such as quality, yield, and stress resistance, are quantitative traits. With the continuous updates and improvements in molecular markers, mapping populations, and statistical analysis methods, quantitative traits locus (QTL) mapping has proved an effective approach for dissecting the genetic mechanisms of complex quantitative traits (Mauricio 2001). In recent years, increasing numbers of candidate genes within QTL intervals for important agronomic traits of crops (tomato, rice, wheat) have been isolated and cloned. In *B. napus*, the first linkage map was a restriction fragment length polymorphism (RFLP) map with 126 sites and a length of 1413 cM was constructed based on a cDNA library explicitly expressed at the seedling stage as a probe (Landry *et al.* 1991). In the subsequent 20 years, many researchers have used germplasm materials with various genetic backgrounds to construct a large number of mapping populations and have applied them to the QTL research of some important agronomic traits in *B. napus* (Udall *et al.* 2006; Zhao *et al.* 2006b, 2012). Until now, a total of 183 plant height-related QTLs in *B. napus* was identified by various markers and explained a phenotypic variation of 3.0–70% (Mei *et al.* 2009; Ding *et al.* 2012; Shi *et al.* 2013; Cai *et al.* 2014; Wang *et al.* 2016, 2020; Huang *et al.* 2020). Traditional QTL mapping typically constructs segregating populations derived from two parental inbred lines, which is laborious, time-consuming, and costly (Salvi and Tuberosa 2005).

With the development of whole-genome resequencing (WGS) and the announcement of the *B. napus Darmor-bzh* reference genome (Chalhoub *et al.* 2014), the density of molecular markers in linkage maps has increased, and the positioning of the QTL is more accurate. *BnDWF1*, a dwarf-dominant locus of *B. napus*, was identified and mapped on linkage group A09 via the construction of a high-density SNP genetic map (Wang *et al.* 2016). Additionally, another method called QTL-seq, which evolved from traditional bulked segregant analysis combined with WGS is widely used in rice and other crops and has been successfully used for positioning QTL or cloning genes involved in disease resistance, flower color, leaf color, flowering time, and yield-related traits (Illa-Berenguer *et al.* 2015; Takagi *et al.* 2015; Singh *et al.* 2016). Thus far, QTL-seq is not only widely used in diploid crops, but is also suitable for polyploids with large and complex genomes (Chen *et al.* 2015). In recent years, a combination of QTL-seq and traditional QTL mapping has become popular for identifying QTL and involves a mutual QTL verification process. In a previous study in *B. napus*, a major QTL underlying boron uptake was identified by QTL-seq and then molecular markers were developed based on resequencing data to fine-map the candidate gene (Hua *et al.* 2016).

To date, a large number of dwarf mutants have been bred in rice, wheat, and other crops. The main mechanism for dwarfing is related to plant hormones, including brassinolide, gibberellin, and auxin. At present, most of the genes that have been cloned in *B. napus* dwarf mutants are involved in gibberellin or auxin signaling pathways (Liu *et al.* 2010; Zhao *et al.* 2017; Li *et al.* 2019; Zheng *et al.* 2019). Here, we constructed two F_2_ populations (Wuchang-2017; Yangluo-2018) that were derived from a cross between two lines with Zhongshuang 11-HP (ZS11-HP) and a natural *B. napus* mutant *sdw-e*. We then performed a joint method of QTL-seq and high-density SNP linkage map construction to identify a major QTL-*qPHA10* for plant height in the two F_2_ populations. Furthermore, based on the variations and the differentially expressed genes (DEGs) in the shoot apical meristem (SAM) tissue of the two parental lines, the putative candidate genes were predicted. Our results will improve the understanding of the genetic and molecular mechanisms underlying *B. napus* plant height as well as provide new molecular markers for breeders.

## Materials and methods

### Plant materials and trait measurements

A natural variation semi-dwarf mutant (*sdw-e*) was a spontaneous mutant that was isolated in a *B. napus* inbred line with normal plant height (about 170 cm) of The Yangtze River Basin in China after continuous selfing eight years. After the selfing of *sdw-e*, the plant and other agronomic traits were inherited stably. F_1_ and reciprocal F_1_ were generated from the cross of *sdw-e* and ZS11-HP. The F_2_ population, which was derived from ZS11-HP × *sdw-e* and shown in Wuchang-2017, was used for QTL-seq. A total of 200 individuals in the F_2_ population that were shown in Yangluo-2018 were used to construct a linkage map for QTL mapping. All the materials were shown uniformly in a row of 2.0-m length with 15 individuals, and the space between the rows was kept at 0.4 m. At maturity, the plant height of the total individuals in the F_2_ population was measured. A total of 10 plants in *sdw-e*, ZS11-HP, and two F_1_ were respectively selected to measure the plant height. All the selected plants were open-pollinated, and plant height was measured from the ground level to the tip of the inflorescence at the mature stage.

### Genetic analysis

Genetic analysis was performed in the F_2_ population (Yangluo-2018) using the mixed major gene plus polygene inheritance model of the SEgregate Analysis (SEA) software (https://cran.r-project.org/web/packages/SEA/index.html) (Zhang *et al.* 2003). Parameters were estimated for each generation (two parents, F_1_, and F_2_) using the maximum likelihood method and the Iterated Expectation and Conditional Maximization algorithm. Then choose the models with the similar lowest Akaike information criterion (AIC) value as the candidate models. And then, the fitness test of the candidate models was performed, including the uniformity test (*U*_1_^2^, *U*_2_^2^, and *U*_3_^2^), Smirnov test (*nW^2^*), and Kolmogorov test (*D*n). According to these fitness tests and AIC value, the optimal genetic model was selected. At last, the first and second order genetic parameters were estimated based on the least-square method.

### WGS library construction and QTL-seq

In the seedling stage, all the individuals of the F_2_ population in Wuchang-2017 were marked and the fresh young leaves of each individual were collected. In the mature stage, the plant height of each individual in the F_2_ population were measured, and the corresponding 20 tallest and 20 dwarf plants were selected in the F_2_ population to construct the two extreme bulks for QTL-seq (Hartwig *et al.* 2012). The high-quality genomic DNA, including two parents and two extreme bulks, were extracted using the Hi-DNAsecure Plant Kit (TIANGEN, Beijing), and paired-end sequencing was performed on the Illumina HiSeq 2500 platform. High-quality clean reads were obtained after filtration of the raw reads, following which the clean reads were aligned to the reference sequence of *B. napus* using Burrows–Wheeler Aligner (parameter: mem -t 4 -k 32 -M) (http://bio-bwa.sourceforge.net/; Li and Durbin 2009; Chalhoub *et al.* 2014). Alignment files were converted to SAM/BAM files using SAMtools (Li *et al.* 2009). The homozygous SNPs were discovered from two parental accessions using GATK software (McKenna *et al.* 2010). According to the sliding window analysis, the SNP-index for all SNP positions in the bulk individuals for the highest and lowest plant height bulk as described previously was calculated (Zhao *et al.* 2020). The Δ (SNP index) was then calculated using the following formula: [SNP index (highest plant height bulk) – SNP index (lowest plant height bulk)]. The distribution of average SNP index and Δ (SNP index) was estimated using a sliding window approach with a 1 Mb window size and 10 kb increment and was plotted to generate SNP index plots for all *B. napus* chromosomes (Takagi *et al.* 2013).

### High-throughput genotyping in the mapping population

A total of 200 individuals in the F_2_ population were subjected to the MGISEQ platform for WGS. Reads filtration and mapping to the *B. napus* reference genome were the same as for the QTL-seq analysis. SNP calling within the mapping population was performed using an in-house pipeline in ‘Sentieon Genomics’ tools. To exclude false variants, the SNPs were filtered by GATK (version 4.1.4.0) based on the parameters: QUAL < 30.0 ‖ MQ < 50.0 ‖ QD < 2.0, cluster size 3, cluster Window Size 10 (McKenna *et al.* 2010). According to the SNP calling method, a total of 677,649 SNPs was obtained. And then, an approach using 15 SNPs as sliding window size and an SNP step size was used to fill and correct the markers. When the number of allelic SNP A:A (or B:B) in the sliding window was not <13, the genotype was considered as A:A (or B:B) and A:B was used for genotype filling and correction in other cases (Huang *et al.* 2009). A bin marker present between two SNPs with no recombination represented one genetic bin.

### Construction of a high-density linkage map and QTL analysis

Bin markers were distributed in the 19 linkage groups of *B. napus*. Taking linkage groups as a unit, HighMap software (http://highmap.biomarker.com.cn/) was used to analyze and obtain the linear arrangement of markers in the linkage group, as well as to estimate the genetic distance between adjacent markers (Liu *et al.* 2014). Centimorgan (cM) distances were calculated by the Kosambi function for map distance (Kosambi 2016). The interval mapping (IM) and composite interval mapping (CIM) methods of the R/qtl software (https://rqtl.org/) was used to analyze QTL mapping (Broman and Sen 2009). The logarithm of the odds (LOD) threshold of the 1000 permutation test value was used for the statistical significance of each QTL using *P *<* *0.01.

### RNA-seq analysis

SAM tissues with two biological replicates in ZS11-HP and *sdw-e* were collected in the bolting stage. Total RNA was isolated using an RNA Prep Pure Plant Kit (TIANGEN, Beijing) and was used to construct the RNA-seq library. The well-constructed libraries were sequenced using the BGISEQ platform. Clean reads were filtered and mapped to the *B. napus* reference genome using SOAPnuke and HISAT software (Kim *et al.* 2015; Chen *et al.* 2018). Bowtie2 was applied to align clean reads to the reference genome followed by counting the rate of gene alignment (Langmead and Salzberg 2012). RSEM was used to calculate the expression levels of genes and transcripts to obtain the fragments per kilo base transcript per million reads (FPKM) values (Li and Dewey 2011). DEGs were identified using the DEseq2 method with parameters of FPKM (fold-change) ≥ 2.00 and adjusted *P*-value ≤ 0.05 (Love *et al.* 2014). The Kyoto Encyclopedia of Genes and Genomes (KEGG) enrichment analysis was conducted using ClusterProfiler and ggplot of Bioconductor (http://bioconductor.org/), an open-source software for bioinformatics.

### Synthesis of cDNA and quantitative real-time PCR analysis

The PrimeScript™ RT reagent Kit with gDNA Eraser (Takara Bio, Beijing, China) was used to generate first-strand cDNA. The relative expression level of the target genes was assessed by quantitative real-time PCR (RT-qPCR) with the *actin* gene of *B. napus* as an internal control following the methods previously described (Zhao *et al.* 2021). The relative quantification of gene expression was calculated using the 2^(−ΔCT)^ method (Livak and Schmittgen 2001). The RT-qPCR results were expressed as the mean ± SD by two biological replicates (each with three technical repeats). The primer sequences are listed in Supplementary Table S1.

### Data availability

Supplementary Figure S1 shows Δ (SNP-index) in QTL-seq on the other 18 chromosomes. Supplementary Figure S2 shows the genotype based on a bin map of 200 individuals in the F_2_ population in Yangluo-2018. Supplementary Figure S3 illustrates a matrix of pairwise recombination values for the F_2_ linkage map using Checkmatrix. Supplementary Figure S4 describes the QTL scanning for plant height on chromosome A10. Supplementary Figure S5 shows Spearman’s correlation plot of the four samples based on the RNA-seq data in the study. Supplementary Table S1 lists the primers used for RT-qPCR. Supplementary Table S2 shows the log-max-likelihood values and AIC values of genetic models in SEA analysis. Supplementary Table S3 shows the fitness test of candidate models in SEA analysis. Supplementary Table S4 shows genetic parameters estimated in the MX1-AD-ADI model. Supplementary Table S5 shows the quality of the sequencing data from the QTL-seq. Supplementary Table S6 shows the statistics of comparison between the sequencing reads and reference genome in the two bulks. Supplementary Table S7 Plant height of 200 individuals in the F_2_ population in Yangluo-2018. Supplementary Table S8 the sequence data quality of 200 individuals in the F_2_ population. Supplementary Table S9 shows the bin marker information and the genotype of the 200 individuals in the F_2_ population. Supplementary Table S10 shows the genetic position of all the mapped Bin markers in each linkage group. Supplementary Table S11 shows the physical position of all the Bin markers in each chromosome. Supplementary Table S12 shows the R^2^ value between the genetic map and the physical map in each linkage group. Supplementary Table S13 shows the quality of the RNA-seq. Supplementary Table S14 shows the DEGs information in the QTL region. Supplementary Table S15 lists the variant information between the two parents in the QTL interval. Supplementary Table S16 lists the candidate genes with missense and nonsense mutations. Supplementary Figures and Tables were available at figshare: https://doi.org/10.25387/g3.14370026.Raw or clean reads generated in this study have been deposited into the CNGB Sequence Archive (CNSA) of China National GeneBank DataBase (CNGBdb) (https://db.cngb.org/) with accession number CNP0001630.

Supplementary material is available at figshare: https://doi.org/10.25387/g3.14370026.

## Results

### Phenotypic identification and genetic analysis of plant height

**Figure 1:**
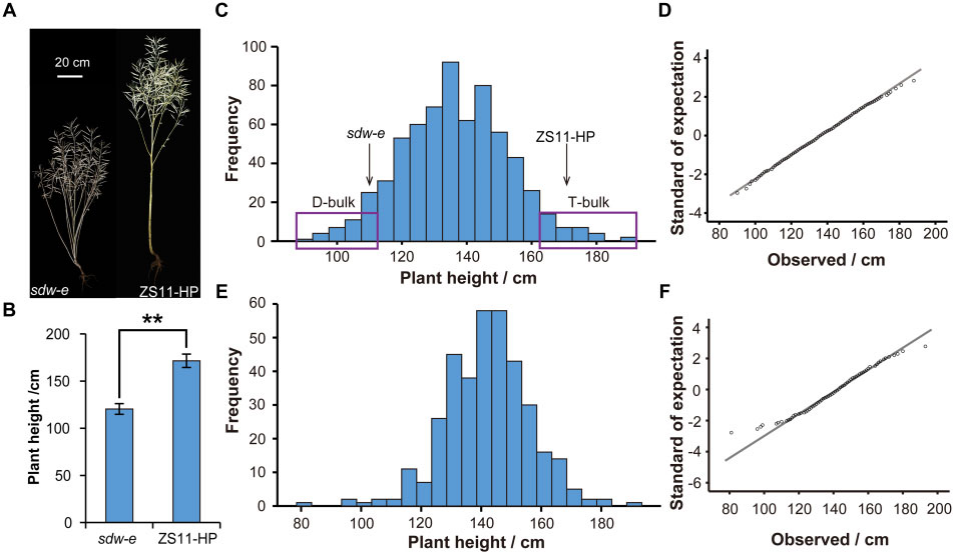
Phenotypic characterization parental lines and F_2_ population. (A) Plant architecture of ZS11-HP and *sdw-e* at the maturation stage. Scale bar = 20 cm. (B) Statistical analysis of plant height between ZS11-HP and *sdw-e*. Thirty plants were measured. Error bars indicate the SD. Significance level of the Student’s *t*-test: **P < 0.01. (C, D) Frequency distribution and expected standard of the F_2_ population in Wuchang-2017. (E, F) Frequency distribution and expected standard of the F_2_ population in Yangluo-2018.

At maturity, ZS11-HP and *sdw-e* clearly differed in plant architecture-related traits, including plant height, branch number, and initial branch height (Figure 1A). In particular, the plant height of *sdw-e* (120.4 ± 5.7 cm) was 30% <ZS11-HP (171.5 ± 7.1 cm; Figure 1B). The plant height distribution frequency of the two F_2_ populations at two sites (Wuchang-2017; Yangluo-2018) conformed to a normal distribution (Figure 1, C–F).

Genetic analysis based on SEA-G4F2 software showed that there were three similar genetic models, including MX1-AD-ADI, MX2-ADI-ADI, and MX2-ADI-AD, of which the model MX1-AD-ADI had the lowest AIC value (Supplementary Table S2). Then, the fitness test of the three candidate models showed that there was no significant difference among them (Supplementary Table S3). These results suggested that the inheritance of plant height in the F_2_ population fitted the MX1-AD-ADI model, which indicated that the plant height was controlled by one major gene of the additive-dominant and additive-dominant-epistatic polygenes. The heritability of the major gene and polygene was 13.9% and 73.1%, respectively (Supplementary Table S4), which suggested that in the F_2_ population the heredity of plant height is greatly affected by the genetic effect of polygenes.

### QTL-seq for the rapid identification of a candidate region

In 2017, we collected 20 extremely tall and 20 extremely dwarfed individuals from the F_2_ population in Wuchang to construct T- and D-bulk, respectively (Figure 1C). WGS of the two parental lines was performed, and about 126.466 Gb raw and 126.234 Gb clean data were obtained, with an average sequencing depth of 69.96 × and 69.62 × and genome coverage of 94.34% and 94.37%, respectively (Supplementary Tables S5 and S6). In the two bulks, a total of 62.364 Gb raw data and 62.195 Gb clean data were produced, and the two bulks (the T- and D-bulk) had an average sequencing depth of 32.82 × and 33.47 × and genome coverage of 96.6% and 96.55%, respectively (Supplementary Tables S5 and S6). All these data suggested that the WGS data were reliable and could be used for further analysis.

**Figure 2:**
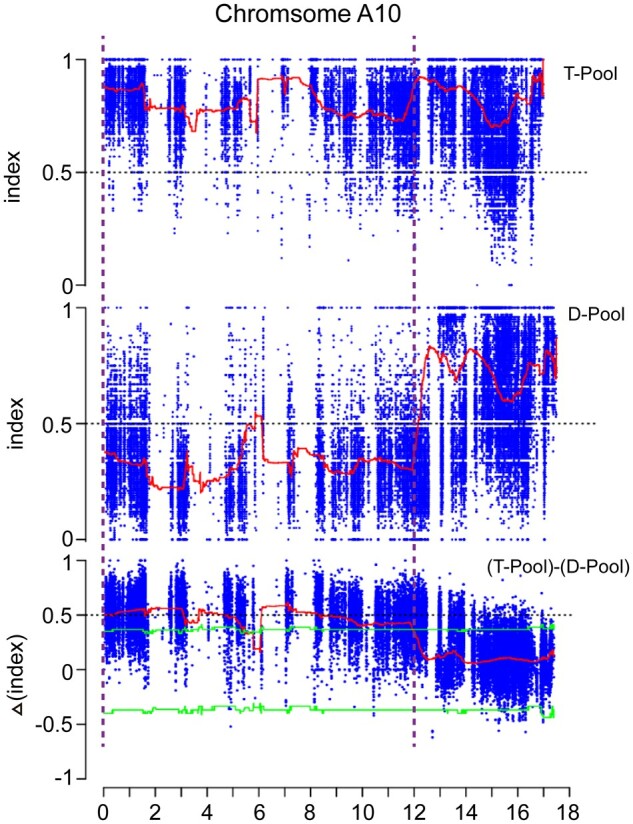
QTL-seq analysis of the F_2_ population in Wuchang-2017. SNP index plot of tall-bulk (top), dwarf-bulk (middle), and ΔSNP-index plot (bottom) of chromosome A10. The red lines represented the distribution of the average SNP index which were estimated using a sliding window approach with a 1 Mb window size and 10 kb step size. The green was the threshold value of 95% for the confidence level.

After filtration and mapping, a total of 936,889 homozygous and polymorphic markers between the two parental lines were obtained and subsequently used to calculate allelic variation frequency (SNP-index) in the two bulks. In QTL-seq, we analyzed the SNP-index of the entire marker sites in the T- and D-bulk with two significant intervals (0–5.5 and 6.0–12.0 Mb) on the A10 chromosome above the confidence interval based on the Δ (SNP-index) method (Figure 2 and Supplementary Figure S1). The QTL-seq showed that the major QTLs for plant height were initially located on chromosome A10.

### Construction and quality evaluation of a high-density genetic map

**Figure 3:**
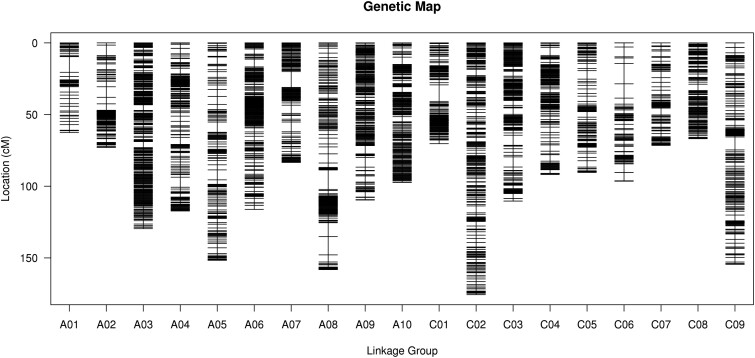
The genetic map constructed by the bin markers.

To construct a high-density linkage map, a total of 200 individuals from the F_2_ population in Yangluo-2018 (Supplementary Table S7) were collected to perform WGS, and a total of 1841.29 Gb clean data were generated (Supplementary Table S8). Among these clean reads, the average quality score of Q20 was over 96.89%, the GC content was about 37.20% (Supplementary Table S8), and the average sequencing depth was 7.67 × for every individual. According to the WGS data of the two parental lines and 200 individuals, a total of 677,649 SNPs were identified, in which 393,214 SNPs were successfully used for map construction. In total, 4,323 bin markers were obtained based on the analysis of recombination breakpoints and were subsequently used for genotyping in 200 individuals (Supplementary Figure S2 and Table S9). All 4,323 bin markers were assigned to 19 linkage groups. Finally, a high-density linkage map with a length of 2026.52 cM was constructed, and the average distance between adjacent markers was 0.47 cM (Figure 3 and Table 1). Of the 19 linkage groups, the longest was C02, reaching 175.64 cM, followed by A08 at 158.11 cM, and the shortest was A01 at only 62.59 cM. The linkage group with the largest number of bin markers was C02 harboring 356 bins, followed by A03 with 345 bins, and linkage group A01 contained the lowest number of bins with only 78 bin markers (Table 1).

**Table 1:** Information on the F_2_ genetic linkage map

linkage group ID	Total bin marker	Total distance (cM)	Average distance (cM)	Max gap (cM)	Gaps < 5 cM (%)
A01	78	62.59	0.8	10.89	98.70
A02	253	72.95	0.29	7.35	99.21
A03	345	129.63	0.38	4.21	100.00
A04	193	117.29	0.61	7	99.48
A05	235	151.63	0.65	7.15	98.29
A06	289	116.17	0.4	5.42	99.65
A07	149	83.39	0.56	10.98	97.97
A08	335	158.11	0.47	13.62	98.80
A09	311	109.61	0.35	6.74	99.35
A10	207	97.32	0.47	5.07	99.51
C01	214	70.35	0.33	11.5	99.53
C02	356	175.64	0.49	4.81	100.00
C03	338	110.36	0.33	7.1	99.41
C04	259	91.81	0.35	5.68	99.61
C05	154	90.36	0.59	5.94	99.35
C06	101	96.48	0.96	14.16	95.00
C07	116	71.52	0.62	4.81	100.00
C08	157	66.86	0.43	4.41	100.00
C09	233	154.45	0.66	9.05	99.57
Total	4,323	2,026.52	0.47	14.16	99.13

**Figure 4:**
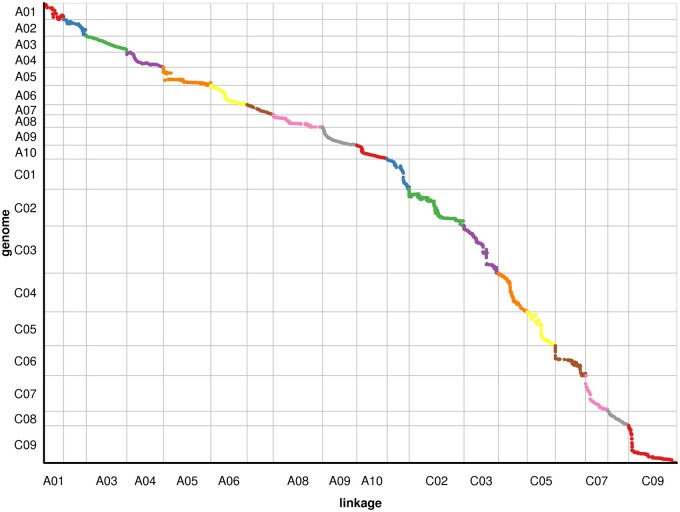
Collinearity analysis of the genetic map and physical map in *B. napus.* The abscissa represents the genetic distance of each linkage group, and the ordinate is the physical length of each chromosome, in which the collinearity of the marker in the genome and genetic map is represented in the form of scattered points.

To check the quality of the genetic map, we analyzed the position of all the mapped Bin markers in the genetic map and physical map (Figure 4 and Supplementary Tables S10 and S11), which showed that the spearman coefficient between the genetic map and the physical map was over 95.8%, suggesting that they had high a collinearity (Figure 4 and Supplementary Table S12). Besides, the matrix heatmap of pairwise recombination values of all mapped bin markers showed that bin recombination was nonrandomly distributed in linkage groups or nonrandomly recombined events in genomes (Supplementary Figure S3). These results suggested that the construction of the genetic map was accurate and reliable.

### Traditional QTL mapping of plant height in *B. napus*

**Figure 5:**
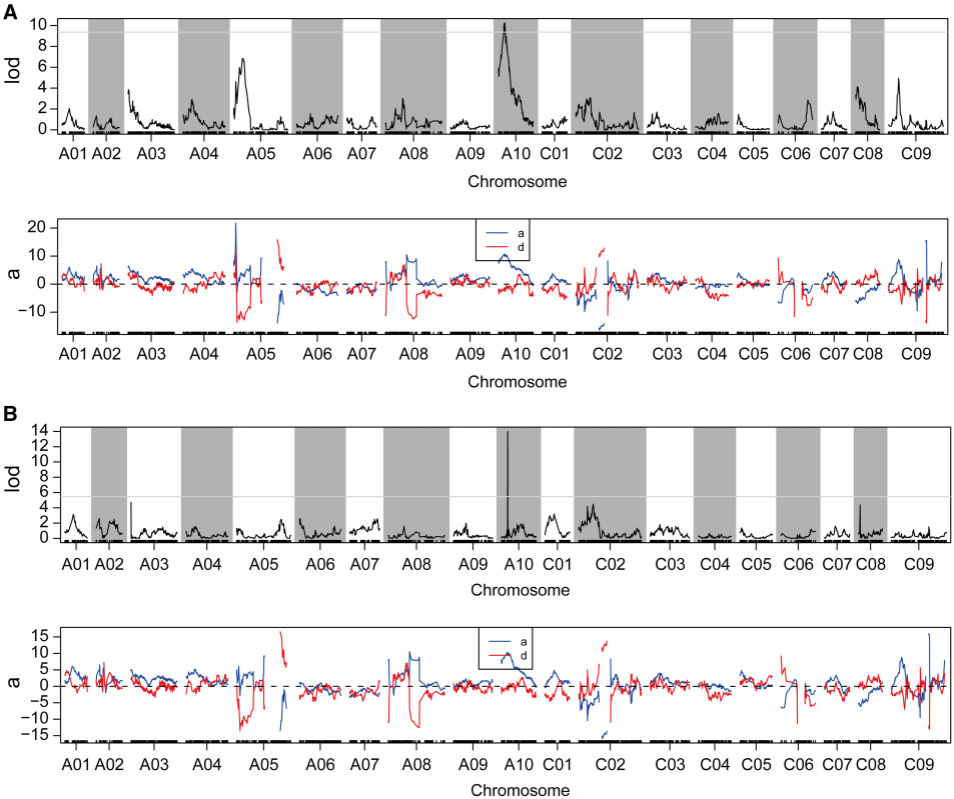
QTL scanning for plant height in whole linkage groups. (A) QTL scanning by IM method. (B) QTL scanning by CIM method. LOD, logarithm of the odds; a, additive effect; d, dominant effect.

According to the high-density linkage map and phenotypic data of the 200 individuals, two QTLs for plant height were mapped on chromosome A10 by IM method (Figure 5A and Supplementary Figure S4A). The QTL with the highest effect was *qPHA10.1-IM-IM*, which had a LOD value of 10.191 and the phenotypic variation explained (PVE) of 18.572%. Another QTL *qPHA10.2-IM* had a smaller effect, with a LOD value of 10.26 and the PVE of 18.482%. The *qPHA10.1-IM* and *qPHA10.2-IM* QTL had an additive effect of 10.705 and 10.676, respectively. The dominant effects of *qPHA10.1-IM* and *qPHA10.2-IM* were −1.453 and −1.505, respectively (Table 2). Both *qPHA10.1-IM* (from 15.02 to 16.77 cM) and *qPHA10.2-IM* (from 17.27 to 19.02 cM) had the same genetic interval of 1.75 cM. In the physical position, *qPHA10.1-IM* (from 2,822,149 to 8,285,330 bp) and *qPHA10.2-IM* (from 4,849,685 to 9,291,804 bp) had an overlapping region, which suggested that the major QTL region identified by IM method consisted of *qPHA10.1-IM* and *qPHA10.2-IM* from 2,822,149 to 9,291,804 bp (Table 2). Based on the QTL mapping by CIM method, the major QTL for plant height named *qPHA10-CIM* was also identified on chromosome A10 with the PVE of 21.528% (Figure 5B, Supplementary Figure S4B, and Table 2). According to the QTL mapping (IM and CIM methods) and QTL-seq, the interval of the major QTL named *qPHA10* for plant height was from 4,849,685 to 8,347,985 bp. Taken together, the major QTL for plant height located on chromosome A10 was identified by QTL-seq and QTL mapping methods in two F_2_ populations from two sites (Wuchang 2017; Yangluo 2018).

**Table 2:** Information of the detected QTL for plant height in the F_2_ population

QTL	LOD threshold	Group ID	Start (cM)	End (cM)	Max LOD	ADD	DOM	PVE (%)	Physical interval (bp)
*qPHA10.1-IM*	9.371	A10	15.02	16.77	10.191	10.705	−1.453	18.572	2,822,149–8,285,330
*qPHA10.2-IM*	9.371	A10	17.27	19.02	10.260	10.676	−1.505	18.482	4,849,685–9,291,804
*qPHA10-CIM*	5.462	A10	17.77	18.02	14.003	10.218	−0.141	21.528	4,849,685–8,347,985

LOD, logarithm of the odds; ADD, additive effect; DOM, dominance effect; PVE, phenotypic variation explained.

### Global and transcriptome difference analysis of gene expression

To study the DEGs for plant height, SAM tissues of ZS11-HP and *sdw-e* were collected from two biological replicates for RNA sequencing. A total of 47.72 Gb clean data were obtained, and the Q30 value of each sample exceeded 95.48%. (Supplementary Table S13). *R*^2^ value represented the correlation based on the FPKM value among samples showed the qualified consistency in biological replicates (Supplementary Figure S5). This confirmed that the transcriptome data were reliable and could be used for subsequent analyses.

**Figure 6:**
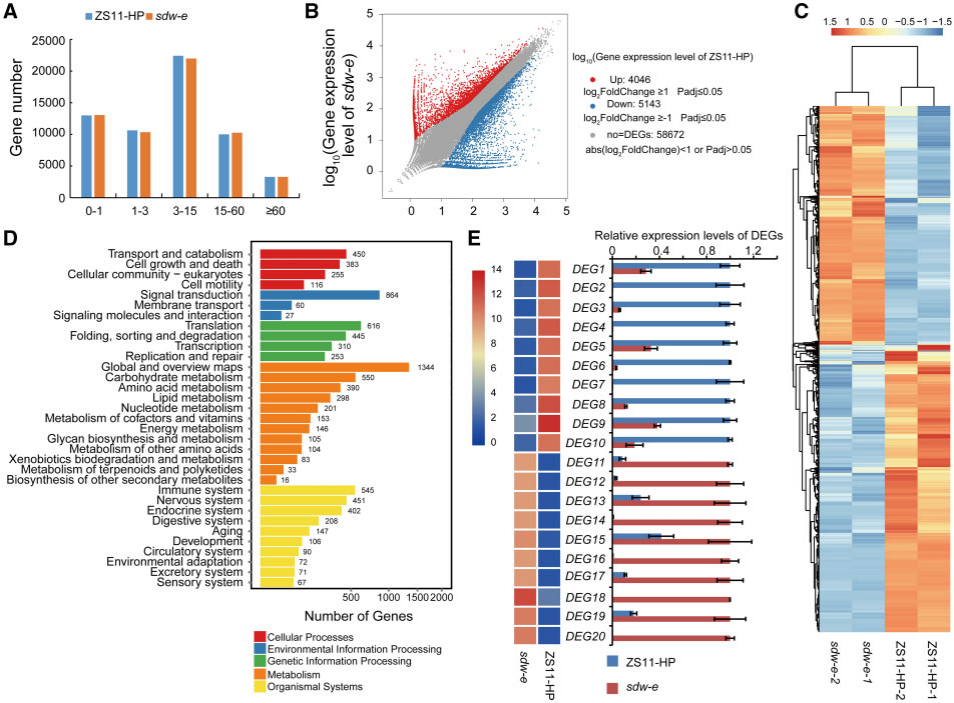
RNA-seq analysis and KEGG enrichment analysis of DEGs. (A) FPKM value distribution of all genes detected in SAM tissues from ZS11-HP and *sdw-e*. (B) The overall alterations and the statistics of DEG numbers in ZS11-HP and *sdw-e*. (C) Heatmap displaying the expression levels of the DEGs in the four samples. The color scale represents FPKM normalized log_2_ transformed counts. (D) The significant KEGG annotations of the DEGs in ZS11-HP and *sdw-e*. (E) Relative expression level verification of 20 DEGs by RT-qPCR. The color scales represent log_2_FPKM.

Based on the FPKM value, 46,334 and 45,847 genes with values of FPKM ≥ 0.1 were identified in ZS11-HP and *sdw-e*, respectively (Figure 6A). Additionally, 21.89% and 22.16% of the genes in ZS11-HP and *sdw-e* had very low expression levels (FPKM < 1.0), 17.94% and 17.56% had low expression levels (1.0 ≤ FPKM < 3.0), 37.78% and 37.80% had moderate expression levels (3.0 ≤ FPKM < 15.0), 16.85% and 17.42% had high expression levels (15.0 ≤ FPKM < 60.0), and 5.54% and 5.56% had very high expression levels (FPKM ≥ 60.0; Figure 6A). Based on the DEseq2 method, a total of 4046 genes were upregulated, whereas 5143 genes were downregulated in the entire genome between ZS11-HP and *sdw-e* (Figure 6, B and C).

**Figure 7:**
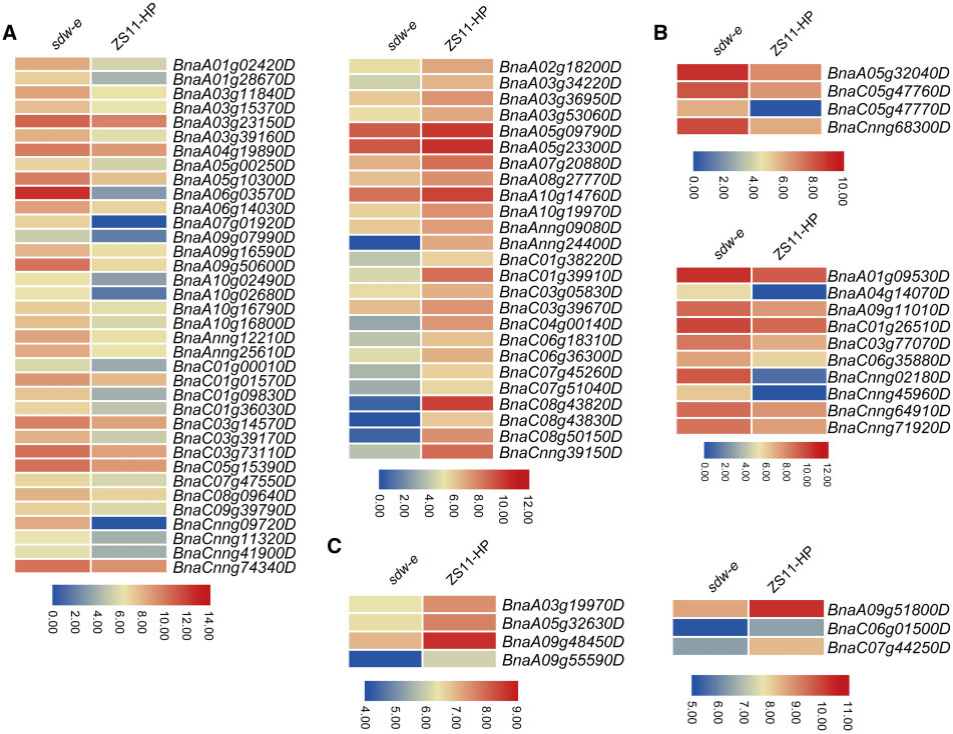
Heatmap of all DEGs in the entire genome involved in phytohormone signaling pathways. (A–C) The expression level of DEGs respectively related to the auxin, brassinolide, and gibberellin signaling pathway. All the color scales represent log_2_FPKM.

To discern the functional distribution of the DEGs, KEGG pathway analysis was performed. The result showed that the most significantly enriched pathways of the DEGs in the SAM tissues were involved in transport and catabolism, signal transduction, translation, global and overview maps (Figure 6D). And then, we analyzed the whole-genome DEGs involved in the phytohormone signaling pathway, which showed that 50 upregulated and 32 downregulated genes were involved (Figure 7), and in which, a total of 61 DEGs (36 upregulated and 25 downregulated) were enriched in the auxin signaling pathway (Figure 7A).

### Candidate gene predicted by integrating WGS and RNA-seq

For further candidate gene excavation, we analyzed the transcriptome file of the SAM tissues in ZS11-HP and *sdw-e*. According to the identified QTL region, a total of 11 upregulated and 14 downregulated candidate genes were identified in the confidence intervals of the stable QTL (Supplementary Table S14). Besides, according to the WGS data of QTL-seq, a total of 1226 homozygous SNPs or InDels were identified between ZS11-HP and *sdw-e* in the major QTL interval, of which most variations were in the intergenic region (Supplementary Table S15). A total of 16 genes with missense and nonsense mutations were identified in the major QTL interval (Supplementary Table S16).

It is known that plant height in *B. napus* is mainly affected by phytohormone biosynthesis and signal transduction pathways (Wang *et al.* 2016; Zhao *et al.* 2017; Li *et al.* 2019). According to the reference genome of *B. napus*, there are a few genes associated with phytohormone metabolism in the major QTL region, including *BnaA10g07740D*, *BnaA10g06890D*, and *BnaA10g06520D* respectively involved in gibberellin, auxin, and abscisic acid metabolism. However, not only the expression level of *BnaA10g07740D*, *BnaA10g06890D*, and *BnaA10g06520D* were no significant difference but also no missense and nonsense mutations occurred in the three genes (Supplementary Tables S14 and S16).

Based on the variation information and RNA-seq in the two parents, missense mutations were identified in *BnaA10g08290D*, *BnaA10g08230D*, and *BnaA10g09290D*, as well as the expression levels of the three genes between the two parents was significant difference (Supplementary Tables S14 and S16), suggesting the two genes could be considered as the candidate genes. Besides, according to the gene functional annotation, *BnaA10g08230D* which encodes a putative tethering factor required for cell plate assembly during cytokinesis, involving in cell wall division was downregulated in *sdw-e* (Supplementary Table S14). Taken together, *BnaA10g08290D*, *BnaA10g09290D*, and *BnaA10g08230D* may be the candidate genes.

## Discussion

### Integrated analysis of QTL mapping and QTL-seq

As assessment of previous studies indicated that the number and distribution of QTL on different chromosomes are inconsistent between different studies, and the contribution rate and effect of QTL with the same trait also differ. The factors influencing QTL positioning mainly include population type, parental variation, molecular marker density, and identification methods. In previous studies, the construction of genetic maps in rapeseed was mainly based on (Simple Sequence Repeats)SSR markers and (Recombinant Inbred Lines) RIL or (Doubled Haploid) DH populations (Cai *et al.* 2012; Wu *et al.* 2013)*.* The construction of RIL and DH populations is a time-consuming process. In recent years, F_2_ populations have been used in the identification of QTL in other species (Zheng *et al.* 2018; Wei *et al.* 2020)*.* With the development of WGS, SNP markers and some other rapid methods have been used to identify QTL regions. In this study, we constructed an F_2_ population with ZS11-HP and *sdw-e*, which had different genetic backgrounds and differed significantly in plant height (Figure 1B). The F_2_ population in Wuchang (2017) was constructed using a high-throughput QTL-seq approach (Figure 2). In Yangluo (2018), we also utilized the F_2_ population to construct a high-density genetic map. The QTL-seq results were highly consistent with the QTL mapping based on the bin map and genetic map (Figure 5). Plant height is a typical quantitative trait with a continuous distribution of phenotypes that are easily affected by environmental conditions. In this study, although we did not analyze multiple sites and years of RIL population data, we did use two rapid methods using the F_2_ population from different years and environmental data, which identified the same major QTL for plant height.

### The selection and utilization of the optimal genetic model based on the SEA software

Previous studies have shown that the effects between genes controlling the inheritance of quantitative traits in plants are not exactly equal, and the interaction of major genes and multiple genes constitutes a major gene-polygene genetic system regulating the inheritance of quantitative traits in plants (Gupta 2002). The establishment of the mixed major gene plus polygenes inheritance model can be used as a general model for the inheritance of quantitative traits in plants (Gai *et al.* 2000). This analysis method can not only evaluate the total genetic effect of plant genes but also analyze the existence of major genes and polygenes and their effect values (Wang and Gai 2001).

In this study, we used two parental lines, F_1_, and the F_2_ population to perform the selection of the optimal genetic model. The result showed three models were similar and the heritability of the polygenes was higher, which suggested that it is a discrepancy with the result of QTL mapping. There maybe two reasons: (1) The generation was not enough. In this study, the F_2_ population, the only segregate population, was used in the SEA analysis. If we’d like to get a more accurate model, backcross or recombinant inbred lines population were needed. (2) The number of plants in the F_2_ population is not enough. (3) In the QTL-seq study for a quantitative trait, one major region in the whole genome was usually identified. In this study of QTL mapping, we only selected the major QTL with the highest LOD value, which was consistent with the result of QTL-seq. Besides, in this study, we also identified other minor QTL, including QTL in chromosomes A05, C08, and C09, which possessed a lower LOD value than the major QTL in chromosome A10 (Supplementary Figure S4). Given the minor-effect QTL, it is also the reason for the existence of several similar models in SEA analysis.

### Construction of a relatively saturated SNP-based genetic linkage map using WGS

The construction of a genetic map is an important procedure in genetic research. It is the basis for understanding and analyzing complex agronomic traits from the molecular level and provides a means for the map-based cloning of important genes. In the genetic map research of *B. napus*, a total of 103 RFLP markers were used for the first linkage map, which covered 1413 cM and assembled into 19 linkage groups (Landry *et al.* 1991). With the development of molecular markers, much more high-density genetic maps have been constructed using amplified fragment length polymorphism, random amplified polymorphic DNA), and RFLP markers covering 2429 cM, which suggests that the density of the markers was saturated and could cover the whole genome at that time (Lombard and Delourme 2001). And then the SSR markers, SNP markers based on SNP Chip, restriction site-associated DNA (RAD) markers, and specific locus amplified fragment (SLAF) markers were used in the construction of genetic map for QTL mapping (Liu *et al.* 2013; Wang *et al.* 2013; Qi *et al.* 2014; Zhang *et al.* 2016, 2019; Chen *et al.* 2017; Zhao *et al.* 2006a). With the development of WGS, a few of genetic maps based on the WGS have been constructed in *B. napus* (Wang *et al.* 2019).

In this study, we constructed a high-density genetic linkage map of *B. napus* using the WGS method and obtained a relatively saturated genetic map that was 2026.52 cM in length and contained 4323 bin markers with an average marker interval of 0.47 cM (Figure 3 and Table 1). In a previous study, based on a 60 K SNP Chip, a linkage map containing 2771 bins with an average distance of 1.47 cM between bins was constructed for *Sclerotinia* resistance and flowering time QTL mapping (Zhang *et al.* 2019). A total of 1329 ddRAD markers were used for the construction of a high-density ddRAD linkage map with a total length of 1610.4 cM (Chen *et al.* 2017). When compared with other markers and methods, nether the length of linkage map or the density of markers, the high-density genetic map based on WGS of this study has higher quality than other markers, including SSR, RAD, and SLAF markers.

### Molecular marker-assisted breeding of plant height

An appropriate reduction of plant height is one of the main objectives of oilseed rape breeding. Using WGS, a large number of SNP markers linked to rapeseed plant height were located in this study. Additionally, two methods applied to data from two sites across 2 years were used to identify the major QTL-*qPHA10*. These results provide reliable and beneficial genetic information for the genetic improvement of plant height in oilseed rape.

Plant architecture is a plastic trait due to the flexibility of plant development. The aboveground plant architecture is formed by meristems that produce new cells and organs. SAM tissue promotes vertical growth (Wang *et al.* 2018). The maintenance of the SAM is of utmost importance for plant growth. Although microscopic observation, correlations of seedling SAM morphology and adult plant phenotypes may render the vegetative SAM predictive of agronomical important plant traits (Thompson *et al.* 2015). Many studies have shown that various plant hormone signals and their synergistic interactions play important roles in the regulation of SAM differentiation, including gibberellin, ethylene, auxin, abscisic acid, and cytokinin (Hamant *et al.* 2002; Shani *et al.* 2006; Gordon *et al.* 2009).

Thus far, in *B. napus*, most of the cloned genes for plant height are involved in gibberellin and auxin signal transduction pathways. The signal transduction of gibberellin is mainly related to the DELLA protein. Both *BnaA06.RGA* and *BnaC07.RGA* encode the gibberellin signal transduction factor DELLA, and missense mutations in the VHYNP domain led to dwarfing in *B. napus* (Zhao *et al.* 2017; Liu *et al.* 2010). The mutations occurred in *BnaA3.IAA7* and *BnaC05.IAA7* and inhibited auxin signaling and caused plant dwarfing (Li *et al.* 2019; Zhao *et al.* 2019; Zheng *et al.* 2019). The development of plant stems depends on the maintenance of SAM at the apex of the growth axis. The petunia HAM encodes the GRAS protein, which regulates the development of lateral organ primordia and stem vascular tissues, and is necessary and specific for the maintenance of SAM (Stuurman *et al.* 2002). In this study of global RNA-seq, most of the DEGs involved in the phytohormone metabolism pathway were enriched in the auxin signaling pathway (Figure 7A). However, in the major QTL interval, no mutation genes were identified related to the phytohormone metabolism pathway. And we predicted three candidate genes, of which *BnaA10g08230D* involved in cell wall division. Further molecular evidence is needed to confirm the associated mechanisms.

## Conclusion

The major QTL for plant height was identified by QTL-seq and a high-density genetic map based on WGS. Moreover, the high-density genetic map was of high quality and was highly saturated. The identified QTL and candidate gene can be used in marker-assisted selection for *B. napus* breeding in the future.

## Funding

This research was funded by the National Natural Science Foundation of China (31770250), National Key Research and Development Program of China (2018YFE0108000), Natural Science Foundation of Hubei Province (2019CFB628), Earmarked Fund for China Agriculture Research System (CARS-12), and Agricultural Science and Technology Innovation Program (ASTIP) of Chinese Academy of Agricultural Sciences.

*Conflicts of interest:* None declared.
